# Menstrual hygiene management in flood-affected Pakistan: Addressing challenges and ensuring women's health and dignity

**DOI:** 10.3389/fgwh.2023.1238526

**Published:** 2023-08-02

**Authors:** Zarnab Tufail, Wania Ahmer, Shanza Gulzar, Muhammad Hasanain, Hussain Haider Shah

**Affiliations:** ^1^ Department of Medicine, Gujranwala Medical College, Gujranwala, Pakistan; ^2^ Department of Medicine, Dow University of Health Sciences, Karachi, Pakistan; ^3^ Department of Internal Medicine, The Aga Khan University Hospital, Karachi, Pakistan

**Keywords:** menstrual hygiene management (MHM), gender-sensitive disaster management, minimum initial service package (MISP) for sexual and reproductive health, floods and menstrual health, menstrual hygiene in Pakistan, menstrual hygiene in emergencies

## Abstract

Pakistan's recent floods have worsened women's and girls' menstrual hygiene problems, compromising their health, dignity, and well-being. Supply chain issues, poor facilities, and cultural stigma limit menstrual products and hygiene management. Gender-sensitive disaster management and menstrual health education programmes can help. The Minimum Initial Service Package (MISP) can provide emergency reproductive health services. Involving men, working with religious leaders, and pre-disaster planning for menstrual hygiene management can help break the taboo and increase access to resources. Meeting ongoing needs requires timely menstrual hygiene product distribution, restocking, and renewal. By addressing these issues, Pakistan can empower post-flood women and girls through economic opportunities and legal protection.

## 1. Introduction

In 2022, Pakistan was hit by recurring floods, causing severe damage to infrastructure, health facilities, crops, and livestock and leaving 6.4 million people in need of humanitarian assistance. Although water levels are receding, 50% of the displaced population still lack access to clean water and sanitation, putting them at risk of disease outbreaks (1). In Pakistan, the floods worsen disparities in healthcare access, particularly between rural and urban areas. Over 1,400 health facilities have been damaged, hindering access to essential healthcare services, medicines, and medical supplies. Among the affected are 650,000 pregnant women and girls lacking support for safe deliveries (2).

**Figure 1:**
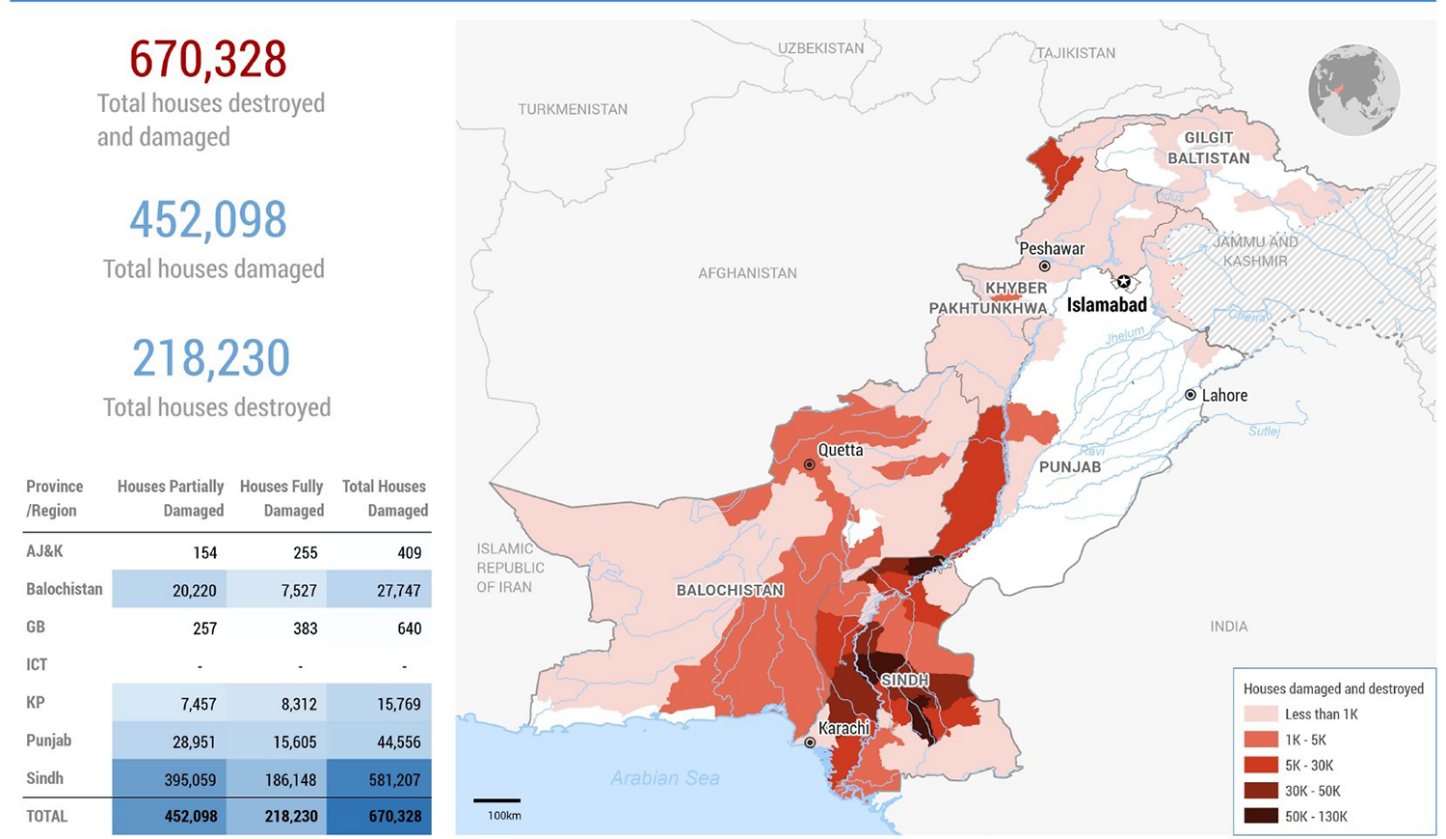
A map released by UNOCHA on August 25, 2022, outlining the flood damage in Pakistan (4).

Even before the floods, Pakistan faced high maternal mortality rates, especially in rural areas. Access to contraception and reproductive health services is also impacted. Additionally, menstruating women face challenges accessing sanitary products, leading to infection risks (3). Figure 1 shows a map released by UNOCHA on August 25, 2022, outlining the flood damage in Pakistan (4).

A study by Azad & A. K (2013) emphasized that these floods disproportionately affect women, with girls being particularly vulnerable during times of disaster (5). They are not only more likely to experience loss of livelihoods, displacement, and an elevated risk of gender-based violence (6) but they also face unique challenges related to menstrual hygiene (5). Therefore, addressing menstrual hygiene becomes even more imperative in order to protect the well-being and dignity of women and girls affected by the floods in Pakistan. Although these factors are significant, it extends beyond the mere availability of sanitary pads and suitable restroom facilities. It encompasses creating an environment where women and girls are valued and supported in maintaining their menstrual management with dignity. This includes promoting gender-sensitive approaches to disaster management, which recognize and address the specific needs and vulnerabilities of young girls and women during and after floods in Pakistan (7).

## 2. Discussion

Pakistan is ranked 145th out of 156 countries in the Global Gender Gap Index Report 2022 for economic opportunity and engagement (8). Floods severely disrupt the supply chain of menstrual products, making them unavailable to women and girls who need them, leading to severe consequences for their health, dignity, and well-being (9–11).

Insecurity, illiteracy, and lack of confidence create barriers to women's employment, particularly during floods when mobility is restricted and access to hygiene products is limited (12). Women in Pakistan often prioritize their family's healthcare needs over their own, resulting in delayed or overlooked medical attention. The Minimum Initial Service Package (MISP) is crucial in delivering essential reproductive health services during crises. MISP is a set of priority sexual and reproductive health (SRH) activities to be implemented at the onset of an emergency that aims to reduce mortality, morbidity, and disability, especially among women and girls (13).

Women in rural areas face challenges accessing sanitary products due to limited familiarity and financial constraints. As a result, they often resort to using washable cloth pads. However, the lack of water, privacy, and suitable drying areas poses difficulties for girls in managing menstrual hygiene, particularly in flood-affected regions. This situation compels women to use unclean materials, leading to health problems such as infections and skin irritations. Inadequate menstrual hygiene management (MHM) can also worsen health issues, including urinary tract infections and reproductive problems (14). Poor menstrual hygiene management (MHM) increases the risk of bacterial vaginosis (BV), which, according to a study, is associated with higher chances of premature delivery, sexually transmitted diseases, and pelvic inflammatory disease (PID) (15).

It can be difficult to address the challenges of menstrual hygiene management in low-income countries during emergencies due to the stigma surrounding menstruation, especially in Pakistan, where girls are often subjected to segregation practices after menstruation begins, and there is a reluctance to discuss the topic openly (16). Additionally, emergency responders may not have the expertise to address the issue effectively (17). In areas affected by floods, the patriarchal culture can make it difficult for women to communicate with primarily male rescue workers. Additionally, distribution venues can be unsafe for women due to large crowds and the risk of sexual assault (17, 18).

In Low middle-income countries (LMICs) like Pakistan, challenges such as insufficient awareness, high costs of sanitary products, and inadequate facilities for MHM negatively impact the health, dignity and participation of girls and women in education and employment sectors. To worsen the situation, menstruation is often stigmatized with a miasma of shame and guilt (19). Not coincidentally, Pakistan has a school drop-out rate of more than 80 per cent for girls and 44 per cent of the girls receive no primary MHM education (20). This gender bias in Pakistan was further highlighted in the aftermath of the devastating floods in 2022, which affected approximately 33 million people (21). Displaced menstruating women are then faced with additional challenges, primarily inadequate access to safe and private spaces for changing and disposal of menstrual products and discomfort in expressing their needs to male health workers (16).

To establish a gender-sensitive response to menstrual hygiene management (MHM) during emergencies, the initial step is to address the stigmatized concepts and implement practical solutions to replace them. Numerous projects led by international and non-governmental organizations like “Aahung” (22) have focused on implementing comprehensive menstrual health education programs and establishing safe spaces where girls can openly discuss menstruation. Several initiatives have developed new sanitary products like menstrual cups and affordable, eco-friendly pads (Couch, 2017). Projects are underway to improve School WASH infrastructure for better menstrual hygiene (UNICEF ROSA, 2012; WaterAid Pakistan, 2018) (17). As most frontline health workers are men, empowering men to talk about menstruation without shame is equally essential. This can be achieved by conducting specialized orientation sessions for frontline humanitarian response workers (23). Organizations can collaborate with religious leaders to help separate the religious views on menstruation and break the taboo surrounding this topic (24).

A study conducted in flood-prone regions of Assam, India, recognizes the importance of pre-disaster planning for MHM and disposal of menstrual waste in collaboration with different departments to design a combined MHM + WASH strategy in developing countries. It also highlights the importance of consulting women about their preferences before distributing menstrual hygiene products (23). One way of implementing this strategy is a pre-emergency database that gathers comprehensive data on local women's menstrual practices and beliefs in disaster-prone locations (16). In order to help organizations and agencies quickly incorporate MHM into their current programming across industries and phases, Columbia University has released a toolkit. This toolkit provides streamlined guidance and support in effectively addressing menstrual hygiene management in post disaster scenarios (25).

The Minimum Initial Service Package (MISP) is crucial in delivering essential reproductive health services during crises (13). It includes reproductive health services, coordination, planning, and essential commodities. MISP should be integrated into disaster preparedness plans and provide necessary equipment and supplies without a needs assessment. Its implementation can prevent sexual violence, reduce maternal and neonatal morbidity and mortality, meet adolescents' needs, combat HIV transmission, and plan comprehensive SRH services in early disaster stages (13). Through MISP, humanitarian actors address immediate reproductive health needs, reduce maternal and newborn risks, prevent unintended pregnancies, and respond to gender-based violence (13). Additionally, the distribution of MHM kits should be timely and include female-led distributors to make communication easier. Information sheets accompanying MHM kits should contain details on menstrual cycle tracking, personal hygiene practices, and instructions for using, maintaining, and disposing sanitary pads (26). To ensure the effectiveness of the strategies, regularly restocking and renewing these resources every month is necessary to meet the ongoing needs.

Azad et al. suggest several measures to empower local women and reduce their vulnerability to any future disasters. These include implementing laws with legal support that can prevent violence against women. Furthermore, a local market system can be established to ensure fair prices for goods sold by flood-affected people, enabling women to afford essential items. Finally, The government and NGOs can introduce local and home-based industries like food processing, cottage, and thread mills to enhance women's economic opportunities. These initiatives can effectively equip women to face and recover from future disasters with improved resilience (5).

## 3. Conclusion

Addressing menstrual hygiene challenges in Pakistan is crucial, especially in the aftermath of the recent floods. It requires comprehensive approaches that encompass education, access to affordable products, safe spaces, and integration of reproductive health services. We can ensure women's and girls’ well-being, dignity, and safety during emergencies by overcoming stigmas, promoting dialogue, and involving various stakeholders. Ultimately, addressing menstrual hygiene challenges is a matter of health and hygiene and a fundamental issue of human rights, equality, and empowerment.

## Data Availability

The original contributions presented in the study are included in the article/Supplementary Material, further inquiries can be directed to the corresponding author.
